# Worse survival in breast cancer in elderly may not be due to underutilization of medical procedures as observed upon changing healthcare system in Poland

**DOI:** 10.1186/s12885-019-5930-8

**Published:** 2019-07-30

**Authors:** Janusz Kocik, Małgorzata Pajączek, Tomasz Kryczka

**Affiliations:** 1 Gerontooncology Department, School of Public Health, Centre of Postgraduate Medical Education, Warsaw, Poland; 2 National Health Fund, Central Office, Warsaw, Poland; 3 Department of Development of Nursing & Medical Sciences, Medical University of Warsaw, Warsaw, Poland; 4 Department of Experimental Pharmacology, Mosakowski Medical Research Center of Polish Academy of Science, Warsaw, Poland

**Keywords:** Breast cancer, Elderly, Healthcare access, Healthcare burden, Undertreatment, Underutilization

## Abstract

**Background:**

Evidence is emerging that older women may tolerate breast cancer therapies equally well as the young ones, providing that they receive good supportive care. It has also been reported that these patients remain outside the current therapeutic standards. The aim of this observational study was to assess the access of breast cancer patients to medical procedures.

**Methods:**

We retrospectively reviewed a database of breast cancer patients registered in the National Cancer Registry in Poland, searching for the numbers of new cases and deaths in the years 2010–2015. We obtained the numbers and costs of key medical procedures provided for these patients from the National Health Fund in Poland. Breast cancer survival in the years 2010–2015 was estimated based on the mortality/incidence ratio. The *t*-Student test and Spearman correlation coefficient were used for the analysis of data obtained from both databases.

**Results:**

There was no increase in survival throughout the years 2010–2015 in both analysed subpopulations of all breast cancer patients below and over 65 years of age, despite an unprecedented rise in healthcare funding in Poland. We noted 37% lower probability of 5-year survival in patients older than 65 years. The average number of outpatient visits and surgical procedures per person per year were slightly, yet significantly (*p* < 0.01), higher in younger vs. older patients (3.9 vs. 3.4 and 1.18 vs. 1.02, respectively). Outpatient chemotherapy was more common in older patients (6.0 vs. 5.25 cycles a year per person on average, p < 0.01). There were no significant differences in the average numbers of hospitalisations for chemotherapy, frequencies of radiotherapy and in the use of targeted therapy programmes (calculated per person per year), between younger and older patients.

**Conclusions:**

Older women with breast cancer are treated similarly to younger patients, but have significantly worse chances to survive breast cancer in Poland. A simple increase in healthcare financing will not improve the survival in the elderly with breast cancer without developing funded individualised care and survivorship programmes.

**Electronic supplementary material:**

The online version of this article (10.1186/s12885-019-5930-8) contains supplementary material, which is available to authorized users.

## Background

**Fig. 1:**
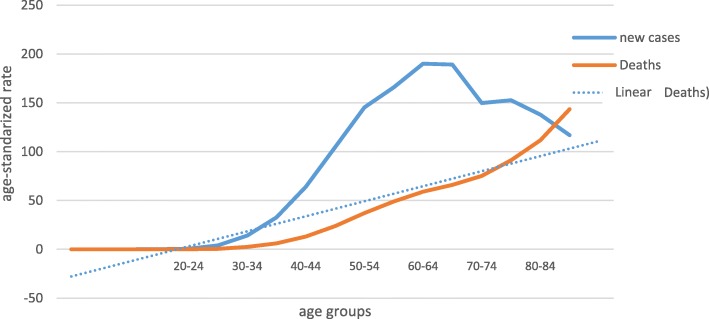
Breast cancer age-related incidence and mortality in years 2010–2015 in Poland

In Poland, breast cancer incidence culminates in younger age groups, but mortality rises linearly with age (Fig. 1). The disease is treated with a standardised sequence of therapy modalities. The standards proposed by international bodies are also recognised in Poland. However, standards for the management in the elderly are lacking due to the inability to collect level 1 evidence. An individual approach is treated with reserve and is based on the extrapolation from studies in the general population or fragmented data from observations in older subgroups.

**Fig. 2:**
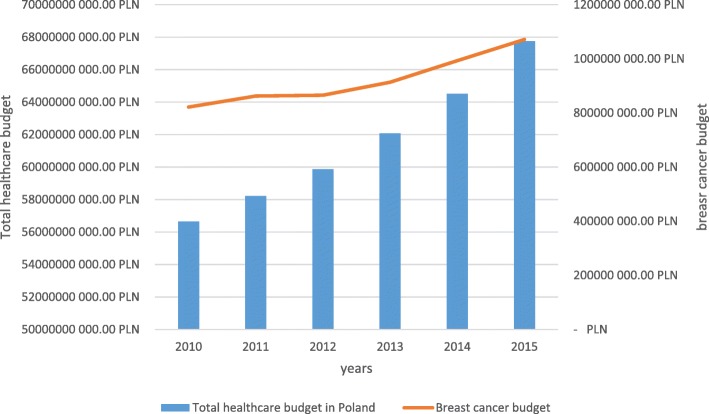
Healthcare funding in Poland 2010–2015

Healthcare funding is continuously growing in Poland and recent years have witnessed an unprecedented growth in the National Health Fund (NHF) budget, including a relatively rapid increase in financing breast cancer treatment (Fig. 2). NHF is the primary, and virtually the only, source of financing cancer treatment in Poland since private insurance covers only some, mainly surgical, procedures. It is also supposed that suboptimal reimbursement costs may be the reason for non-compliance with guidelines [1]. As it has been shown elsewhere, not only the differences in healthcare systems between countries at different levels of socio-demographic development, but also the socioeconomic status of patients within the healthcare system of a given country may influence breast cancer survival [2].

In some countries, new and expensive therapies are limited to selected groups of patients, particularly if there is little data on their clinical benefit. The inclusion criteria do not include age, but they limit treatment to otherwise relatively healthy patients with a good prognosis and with no severe comorbidities, without defining severity in detail. General performance, assessed with popular screening tools such as ECOG, WHO or Zubrod scales, is one of the primary criteria. Older and low-performing patients are frequently excluded without attempts to verify whether there are any reversible causes of their poor general status. Underfunding results in an underdevelopment of social care in multidisciplinary teams, active treatment and palliative care are segregated due to the separation of financing.

The use of surgery, radiotherapy and pharmacotherapy in breast cancer depends on the clinical and pathological stage of the disease that is associated with the risk of recurrence and, consequently, death from cancer. Predictive factors are used in drug selection for hormone therapy and, recently, for targeted therapy. The standards are built on the results of randomised trials. There is still little data on treatment outcomes in the elderly. This may lead to hesitation and neglect in the use of more aggressive, toxic or expensive therapies in older patients, who may be frail and usually have several concomitant diseases. These patients may not gain any clinical benefit from the treatment, but departing from guidelines towards undertreatment may also lead to poor clinical outcomes [3–5].

On the other hand, it is known that the biology of breast cancer may be favourable in older women [6]. With this in mind, there is a belief that resigning from some aggressive or toxic interventions, which have been shown to effectively prolong survival in younger patients, does not affect treatment outcomes in older ones. We intended to verify these hypotheses based on a large number of cases and medical procedures, which are usually provided in the primary treatment of breast cancer within the framework of a uniform health care system. We compared the number of all procedures and the use of treatment modalities between patients above and below 65 years of age in the years 2010–2015 in Poland.

## Methods

The crude numbers of all new cases and deaths in Poland in different age-groups in the years 2010–2015 were obtained from the National Cancer Registry. The data are available at the Registry website: http://onkologia.org.pl/raporty/#tabela_nowotwor_wg_wieku (access: July 23, 2018). The numbers of outpatient visits in oncology clinics, surgical procedures for cancer, outpatient chemotherapy cycles, hospitalisations for chemotherapy and radiotherapy as well as cycles of targeted therapy (trastuzumab only in the period analysed) were obtained from the National Health Fund (with the courtesy of Małgorzata Pajączek, MSc, co-author). Virtually all key medical procedures provided for new breast cancer patients in Poland are registered by the National Health Fund. Total public healthcare costs were compared to breast cancer treatment costs and the growth rate was calculated. The numbers of particular procedures per person were calculated for age groups above and under 65 years. Changes in chemotherapy settings and the resulting shift of costs from inpatient to outpatient chemotherapy were shown. Survival was estimated with the use of mortality-incidence ratio complement (1- M/I ratio), where: M = mortality and I = Incidence. The equivalence of this measure to the actual 5-year survival measured in the population sample was shown elsewhere [7, 8]. MIR complement was calculated for the group above 65 years vs. the group below 65 years of age. As the data represent virtually the entire population and all interventions in the analysed population, basic descriptive statistics were used. A standard deviation for the means was calculated. The means were checked for a true difference using the *t*-Student test for independent variables. A correlation between the relative survival measure and the number of procedures per person in respective age groups was measured with the use of Spearman correlation coefficient (Statistica ver. 13.0, StatSoft, USA).

## Results

**Fig. 3:**
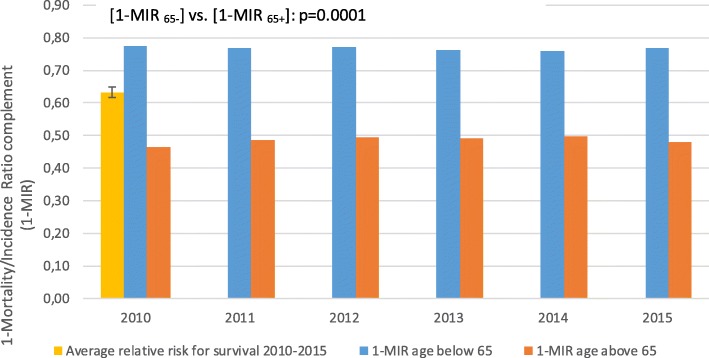
Breast cancer survival in Poland in 2010–2015 estimated by 1-Mortality/Incidence Ratio (1-MIR) - comparison between age groups above and below 65 years

**Fig. 4:**
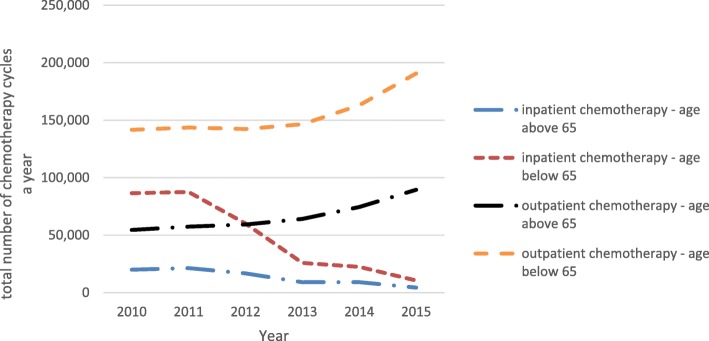
Changes in chemotherapy settings in age groups above 65 and below 65 years in period of 2010–2015 in Poland

A clear growth in the NHF budget has been noted, including the funds for breast cancer treatment. The part of the total healthcare budget that is allocated to breast cancer treatment varied around 1.5% and slightly rose from 1.45% of total NHF expenditures in 2010 to 1.58% in 2015. In the same period, the overall 5-year survival in both age groups, i.e. below and above 65 years of life, remained almost at the same level. Mean survival rates were 77 ± 1% and 49 ± 1% for the younger and older age groups, respectively. Alarmingly, they were found to be systematically and significantly decreasing in older patients (*p* = 0.0001). The relative risk [a proportion of MIR complements (1-MIR) for the groups above and below 65 years of age] was 63 ± 2%. The older group had 37% lower probability to survive after a breast cancer diagnosis (Fig. 3). The average number of outpatient visits per person was slightly, but significantly, higher in the younger vs. older group (3.9 vs. 3.4 visits per person per year, *p* < 0.01, see Table 1). The frequency of surgical procedures in both groups was about 1 (1.18 vs. 1.02 in younger vs. older patients, *p* < 0.01, Table 1), indicating that the patients usually had one breast cancer surgery in their cancer history. Some of them had additional surgeries most probably related to resection of recurrences and axillary lymphadenectomy or sentinel node biopsy. Radiotherapy was used slightly more than once in cancer history in almost all cases, but the frequency of the procedure was comparable between the age groups (1.37 vs. 1.34, younger vs. older group, *p* = 0.77, Table 1). Repeated radiotherapy was most probably associated with axillary irradiation or, to a lesser extent, recurrence irradiation. Outpatient chemotherapy was more frequent among older patients (6.0 vs. 5.25 cycles a year per person on average in older and younger age groups, respectively, *p* < 0.01, Table 1). The rates of hospitalisation for chemotherapy were almost the same in both age groups (3.2 vs. 3.0 cycles a year per person on average in older and younger age groups, respectively, *p* = 0.53, Table 1). A consistent transition to outpatient chemotherapy setting was noticed in the analysed period (Fig. 4). There was no difference between the use of targeted therapy programmes (only trastuzumab in the analysed period) between the age groups. Trastuzumab was used on average in 8.7 vs. 8.6 cycles per person per year in the years 2013–2015 in older vs. younger patients, respectively, *p* = 0.90, Table 1).

**Table 1:** Comparison of average numbers of procedures per patient in 2010–2015, between 65- and 65 + women

Procedures	65-	65+	*p*-significance
Outpatients visits	3.95	3.43	*p* < 0.01
Surgical procedures	1.18	1.02	*p* < 0.01
Outpatient chemotherapies	5.25	5.98	*p* < 0.01
Inpatient chemotherapies	3.22	3.03	*p* = 0.53
Radiotherapies	1.37	1.33	*p* = 0.77
Targeted therapy procedures	8.73	8.64	*p* = 0.90

We found no significant correlation between MIR complement, as a reflection of survival, and the use of any particular medical procedure in older and younger age groups (Table 2).

**Table 2:** Statistical correlations between MIR complement [1-MIR] and treatment procedures

Procedures	1-MIR (65-)	1-MIR (65+)
Average no. of outpatients visits per patient	0.6571	-0.2571
Average no. of surgical procedures per patient	-0.3142	0.4285
Average no. of outpatient chemotherapies per patient	-0.2000	-0.3142
Average no. of inpatient chemotherapies per patient	0.5428	-0.3714
Average no. of radiotherapies per patient	0.2571	-0.4285
Average number of targeted therapy procedures per patient	-0.7142	0.0285

## Discussion

We conducted an observational comparative study to assess the use of medical procedures in the population of breast cancer patients in the age groups below and above 65 years of age in the years 2010–2015 in Poland and their association with survival. There is a continuous debate on the appropriate age of distinction between older and younger population of breast cancer patients. Although in the developed countries this discrimination tends to be shifted to 70 years of age, we adhered to the WHO recommended age of 65 years of life, as the biological age of Polish women is higher compared to the Western Europe, as well as both life expectancy and healthy life years are shorter [9]. We have shown an alarming 37% difference in breast cancer patient survival between older and younger groups. There was no change or an insignificant trend towards improved survival in both groups in the analysed period. No or limited improvement in survival rates for older patients with breast cancer over the last decades was also shown in other studies [10, 11].

Worse survival in aged breast cancer patients was consequently shown in several studies in the last years. It was unequivocally associated with the limited use of the available treatment modalities in older patients [3, 5, 12, 13]. We have shown significant differences in the frequency of use of some procedures between both age groups, however these differences were small or insignificant. 

Mortality registries may be biased by over-reporting cancer as a death cause, whereas in fact other disease entities may be the true reason, with cancer being only a trigger. Indeed, older women with breast cancer and concomitant diseases are shown to have 20 times higher risk of death from reasons other than cancer [14]. In the case of aged patients, there are many possible competitive direct causes of death, including exacerbation of concomitant diseases by cancer itself and treatment toxicity, often indistinguishable for the reporting physicians.

We have not demonstrated any influence of the healthcare budget for breast cancer or the resulting number of procedures on survival in both age populations, even at the end of the analysed period (Table 2). First, the rise in the number of procedures is a direct consequence of the growing incidence of breast cancer. Secondly, the deaths in the particular years of the analysis were a consequence of many variables during several preceding years, including a positive change in healthcare financing and accessibility. There is still a chance that this improvement will result in longer survival in the following years.

The limited use of treatment interventions appears to be the cause of worse survival in older patients. Women older than 75 years were reported to receive a less intensive treatment more than a decade ago. A total of 32% of these patients were assigned to endocrine therapy (tamoxifen) only. About 33% were subjected to mastectomy. BCT and adjuvant treatments were offered to 14% [12]. In this study, 51% of women treated with tamoxifen alone survived 5 years (HR 0.4; 95% CI: 0.2–0.7), while 5-year survival reached 90% (HR 0.1; 95% CI: 0.03–0.4) in the group subjected to BCT and adjuvant treatments.

There is still a poor consensus in the oncology community on the optimal mode of management in older patients with breast cancer. The controversy that exists is whether undertreatment in the elderly really results in adverse outcomes. The statistics in our observational study show a large disparity in survival in elderly breast cancer patients compared to those younger, and only slight differences in healthcare use.

It has been shown that a failure to fully adhere to standards in the elderly undoubtedly reduces their lifespan. However, the standards are also being currently improved to achieve the right balance between the toxicity of aggressive therapies and the clinical benefits for all breast cancer patients. Opinions are expressed that different therapeutic goals should be set for older patients and that life prolongation is as important as the quality of the remaining life and the tolerability of therapy. In fact, long-term adverse events of cancer disease and applied therapies, which contribute to age-related disabilities, exacerbate comorbidities, leading to delayed deaths. These fatalities may be reported as not related to cancer itself. Thus, we argue for balanced and personalised approaches in older breast cancer patients that may improve their cancer survival without further stretching their already limited vital reserves.

### Study limitations

Due to the low granularity level of data in the National Health Fund registry, we were unable to make any detailed conclusions on the influence of any type of treatment modality on the differences in survival in the analysed groups of patients. We had no knowledge on the range, intent, sequence (primary or recurrent) or completion of surgery or radiotherapy. We were unable to analyse the settings of chemotherapy or hormone therapy (adjuvant or palliative) and patients’ adherence to the treatment. The conclusions on the appropriateness of pharmacological therapies are limited. Despite above, we still believe that the comparison of the number of visits for different types of services that must have been provided to be registered by the NHF for reimbursement is sufficient for conclusions. A better characterisation of the problem would require randomised trials. Although this type of study setting leaves behind some important data, it shows the actual performance of the healthcare system and reflects the feasibility of standards followed in the general community better than randomised controlled studies [15].

## Conclusions

Mortality/Incidence ratio (MIR) is a measure that was previously presented by others as associated with healthcare funding and this relationship corresponded in some cases to the outcomes of cancer patients [8, 16]. We have shown that the survival may not be strictly associated with the level of healthcare funding since there was no increase in survival at the end of the period of a clear rise in healthcare funding in Poland. Therefore, it seems that not only the level of funding, but also the integrity and quality of healthcare influence the survival in the most sensitive patient populations. Differences in the number of treatment visits by elderly and younger women with breast cancer in Poland were small at the level of the entire healthcare system if compared e.g. to the large disparities between young and older patients with CNS tumors (data not shown). Nevertheless, there is still a worrying gap in the survival between young and older breast cancer patients, despite the fact that the healthcare burden incurred by the disease is similar in both age groups. Therefore, it seems that further optimisation of cancer therapies to improve toxicity profile and compensate for comorbidities is a better option for elderly patients than adopting the standards for the general population. Our study also shows that a simple increase in healthcare financing will not improve the survival in older patients with breast cancer without developing funded individualised care programmes supported by specialised teams of oncologists, geriatricians, nurses and social workers collaborating under well-tailored survivorship programmes.

## Additional file

Additional file 1: ‘Breast cancer in Poland - National Cancer Registry and National Health Fund data – 2010-2015’. The Supplementary file contains data: a) the numbers of all new cases and deaths in Poland in the particular age-groups (due to breast cancer); b) the numbers of: outpatient visits, surgery procedures, outpatient chemotherapy cycles, hospitalisations for administration of chemotherapy, hospitalisations for administration of radiotherapy, hospitalisations for administration of targeted therapy; data were calculated in total and per patient; c)health care funding in years 2010–2015 in Poland; d) the breast cancer survival in the particular age-groups in the years 2010–2015 estimated with the mortality-incidence ratio complement (1- M/I ratio). (XLSX 20 kb)

## Data Availability

All data generated or analyzed during this study are included as its Additional file 1.

## Abbreviations

BCT: Breast conserving therapy
MIR: Mortality/incidence ratio
NHF: National Health Fund
